# Elastic Textile Wristband for Bioimpedance Measurements

**DOI:** 10.3390/s23063351

**Published:** 2023-03-22

**Authors:** Giuseppina Monti, Federica Raheli, Andrea Recupero, Luciano Tarricone

**Affiliations:** 1 Department of Engineering for Innovation, University of Salento, 73100 Lecce, Italy; 2 CNIT—National Inter-University Consortium for Telecommunications, 43124 Parma, Italy; 3 Independent Researcher, 73100 Lecce, Italy

**Keywords:** bioimpedance, textile electrodes, wearable

## Abstract

In this paper, wristband electrodes for hand-to-hand bioimpedance measurements are investigated. The proposed electrodes consist of a stretchable conductive knitted fabric. Different implementations have been developed and compared with Ag/AgCl commercial electrodes. Hand-to-hand measurements at 50 kHz on forty healthy subjects have been carried out and the Passing–Bablok regression method has been exploited to compare the proposed textile electrodes with commercial ones. It is demonstrated that the proposed designs guarantee reliable measurements and easy and comfortable use, thus representing an excellent solution for the development of a wearable bioimpedance measurement system.

## 1. Introduction

Bio-impedance analysis (BIA) is a low-cost and non-invasive method widely adopted for body composition and clinical condition assessments [1,2,3,4].

The electrical properties of human tissues are exploited to estimate the fat mass and fat-free mass of an individual, starting with body impedance measurements.

There are numerous applications that can benefit from BIA [1,2,3,4,5,6,7,8,9,10,11,12,13,14,15]: cardiovascular monitoring, monitoring dialysis and cancer patients, and monitoring the hydration of athletes during training and competitions.

For some of these applications, particularly in the case of hydration monitoring, a real-time and continuous monitoring is useful.

Accordingly, in the recent literature, some studies have investigated the possibility of replacing traditional BIA electrodes with textile electrodes.

These studies take advantage of the enormous progress in the field of wearable electronics, from the use of conductive textile materials or materials that guarantee the same wearability as a common fabric to the development of extremely compact electronic modules [15,16,17,18].

The main problem that must be addressed in the development of wearable devices is the need to ensure comfortable and practical use. To this end, a device can be integrated into common wearable accessories (watches, bracelets, belts, etc.) or into clothing using non-conventional materials.

Referring to the development of wearable electrodes for BIA, the main problem to overcome lies in the difficulty of developing textile solutions capable of providing reliable measurements without the use of liquid/gel moistures to reduce the contact resistance between the electrode and the skin [18,19,20,21,22,23,24].

In [12], a multi-segment and multi-frequency bio-impedance monitoring platform was presented. The proposed system was able to detect bio-impedance changes associated with fluid shifts due to physical exercises or postural changes. The focus was on the development of compact and wearable electronic circuitry that provided current excitation and voltage measurements, while the adopted electrodes were commercial disk electrodes similar to those used for electrocardiograms.

In [20], two dry-textile electrodes for bioimpedance spectroscopy measurements were presented. The ankle-to-wrist whole body measurements of three subjects in the frequency range of 3 kHz to 500 kHz were analyzed. It was demonstrated that the proposed textile electrodes produced constant and reliable bioimpedance spectra. Regarding the agreement with the commercial electrodes, the error as function of the frequency was analyzed, and a certain deviation at high frequencies was observed.

In [21], fully textile electrodes for hand-to-foot whole-body bioimpedance measurements were presented and compared with conventional Ag/AgCl electrodes. A configuration with four rectangular electrodes was investigated. The measurements of three subjects in the frequency range of 3–500 kHz were reported and discussed. The reported results showed stable and reliable measurements that were better than those reported in the reference literature due to the optimization of the straps. 

In [22], five different types of textiles as band electrodes for calf bioimpedance measurements were investigated and compared with conventional Ag/AgCl electrodes. The measurements of ten subjects in the frequency range of a few kHz up to 1 MHz were analyzed. The achieved results demonstrated that band electrodes allow for obtaining a more uniformly distributed current, and thus, they are preferable to spot electrodes for segmental measurements.

The results presented in [20,21,22], even though few subjects were analyzed, not allowing for a complete statistical analysis, demonstrated the feasibility of using textile materials for the electrodes and the convenience of continuing with studies on the subject.

In [23], textile electrodes fabricated by using the Silitex P130 fabric by Statex were presented and discussed. The Silitex P130 is a bi-elastic silver-plated knitted fabric with a layer of conductive silicone on one side. The proposed electrodes were rectangular electrodes with an area similar to that of a disposable commercial electrode, and the presence of the silicone layer allowed for reduced contact resistance, even in the absence of liquid moistures. However, the measurement campaign showed the need to pay great attention to the application of the electrodes, especially in the presence of hair. 

An interesting and new implementation of electrodes was proposed in [24], where bracelet-shaped electrodes fabricated by using a textile-conductive fabric were presented.

In this paper, the preliminary results presented in [24] are further investigated and discussed. Various implementations of the proposed bracelet electrode are analyzed, and the results obtained from 40 subjects are discussed. It is shown that the proposed electrodes exhibit an excellent correlation with Ag/AgCl commercial electrodes and provide stable and reliable measurements, thus representing a good solution for developing a wearable system for bioimpedance measurements.

The paper is structured as follows: In Section 2, after a brief introduction to BIA, the proposed textile electrodes are illustrated. Experimental results are presented and discussed in Section 3, and conclusions are drawn in Section 4.

## 2. Materials and Methods

### 2.1. BIA in Brief

BIA is a non-invasive method for body composition evaluation from impedance measurements of the human body. A small AC (alternating current) current is applied, and the corresponding voltage drop is measured. As a result of the ratio of the measured voltage drop and the applied current, a complex impedance is obtained:

$$Z=\frac{V}{I}=R+jX.$$

The resistive part of *Z* and *R* is mainly due to the amount of total body water, while the reactive part, *X*, is related to the capacitance of the cell membranes [3]. In most cases, the representations of *Z* in terms of module, |*Z*|, and phase angle, PA, are used. Bioimpedance measurements and, in particular, the phase angle, play key roles in the assessment of an individual’s clinical condition.

In fact, biological tissues exhibit a complex electrical impedance that depends on tissue composition, health status, and the frequency of the applied AC current. The human body consists of biological tissues which, in turn, consist of cells that contain intracellular fluids and are suspended in extracellular fluids. Both intracellular and extracellular fluids have high conductivity (low resistance), being composed of ionic solution and other highly conducting materials. Intra- and extra-cellular fluids are separated by the cell membrane, which consists of a nonconducting layer sandwiched between two conducting layers. Accordingly, the cell membrane behaves as a capacitor, and therefore, it exhibits a reactance that decreases as the frequency of the excitation current increases. Consequently, the ability of the applied current to penetrate the cells depends on its frequency. At low frequencies (1–20 kHz), the current path is concentrated outside the cells and is more affected by extracellular fluids. For higher frequencies (20–200 kHz), the current path involves both extracellular and intracellular fluids.

With the above considerations in mind, analytical formulas that exploit the measured values of *R* and *X* and anthropometric parameters to estimate the body composition have been proposed in the literature. In this regard, various models which differ in the number of compartments used to calculate the body composition are available. In the simplest one, the body is divided into only two compartments: fat and fat-free mass. In more sophisticated models (multi-compartment models), the following main compartments are adopted:

- Total body water (TBW): This is the sum of intra-cellular water (ICW) and extra-cellular water (ECW).
- Extra-cellular water (ECW): This is fluid localized outside cells. ECW is a very important parameter for evaluating a person’s hydration status since most of the fluids lost through sweating come from extracellular compartments. Given that changes in intracellular fluids are usually very small (typically less than 5%), most changes in TBW are due to changes in extracellular fluid. The evaluation of these compartments makes it possible to identify problems related to water retention, malnutrition, or local inflammation, which can lead to high ECW values.
- Fat-free mass (FFM): This refers to all body mass that is not fat. With respect to fat mass, FFM has a higher conductivity due to its higher water content. Various formulas are available in the literature to evaluate FFM using bioimpedance measurements. The simplest formulas calculate the FFM starting with the height of an individual and the measured resistance *R*. More complicated formulas have recently been proposed, and these formulas also exploit other parameters, such as, for example, weight, age, sex, measured reactance, and anthropometric parameters.
- Body cell mass (BCM): This represents the metabolically active tissues of the body. The BCM is an important index for assessing the physical condition of an individual, and it tends to decrease with age or due to poor nutrition. BCM is used to evaluate nutritional status in hemodialysis patients, and in general, it is a good reference value for the calculation of nutrient needs.
- Fat mass (FM): This is the fat mass calculated as the difference between the total weight and the FFM. With respect to FFM, FM has a lower water content and thus a lower conductivity.

### 2.2. BIA Measurements

Various instruments and methods are available for body composition assessments from impedance measurements. As already mentioned, a key role is played by the frequency of the applied AC currents and instruments for single-frequency or multi-frequency measurements. BIAs based on single-frequency measurements exploit empirical linear regression models for bioimpedance values measured at 50 kHz. Multi-frequency approaches use mathematical models and mixture equations based on bioimpedance measurements taken at different frequencies (0, 1, 5, 50, 100, and 200 to 500 kHz).

Regarding the way measurements are performed, most of the commercially available instruments perform a whole-body impedance measurement by using four electrodes, where two electrodes are used for injecting the current and two are used for measuring the voltage drop. Different placements can be adopted as follows:

- Hand-to-foot (H-F): In this case, two electrodes (one for the current and one for the voltage) are applied to the hand and two are applied to the foot.
- Hand-to-hand (H-H) [1,2,3]: In this case, one pair of electrodes is applied to one hand (one for the current and one for the voltage) and the other pair is applied to the other hand.

In both the H-F and H-H placements, the distance between the current electrode and the voltage electrode is a few centimeters, and starting from the end of the body, the current electrode precedes the voltage electrode.

More recently, segmental-BIA approaches have been proposed. In these approaches, the impedance of a portion of the body is exploited for estimating abdominal fat, fluid accumulation in the pulmonary or abdominal region of the trunk, etc. However, for these approaches, standard protocols for the type and the placement of electrodes are not yet available.

### 2.3. Setup Adopted for the BIA Measurements

The BIA 101 analyzer by Akern, compliant with Directive 93/42 CEE and Standard CEI EN 60601-1 (1998/12), was used for measuring the bioimpedance [23,24,25]. The instrument performs single-frequency measurements at 50 kHz, and the output is the impedance in terms of real and imaginary parts. The BIA analyzer is equipped with a calibration kit that ensures a measurement accuracy equal to ±10 Ohms for |*Z*| and ±0.9 degrees for the phase angle.

**Figure 1:**
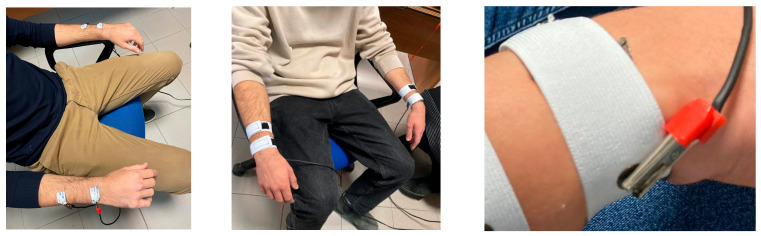
Setup adopted for the measurements with commercial (**left**) and textile electrodes (**middle** and **right**).

Total body H-H measurements were performed on participants (the persons under test, or PUTs) in a sitting position, as shown in Figure 1.

Forty healthy PUTs volunteered to participate in the study. The volunteers were Italian men and women aged between 19 and 26 years. Each of them was informed and provided written and oral consent to participate. The characteristics of the PUTs (age, height, weight, and gender) are provided in Table 1.

For each PUT, reference measurements were performed with commercial Ag/AgCl electrodes (with a contact area of 4.4 cm^2^) produced by EF Medica Ltd., Caldaro, Italy.

### 2.4. Proposed Textile Electrodes

**Figure 2:**
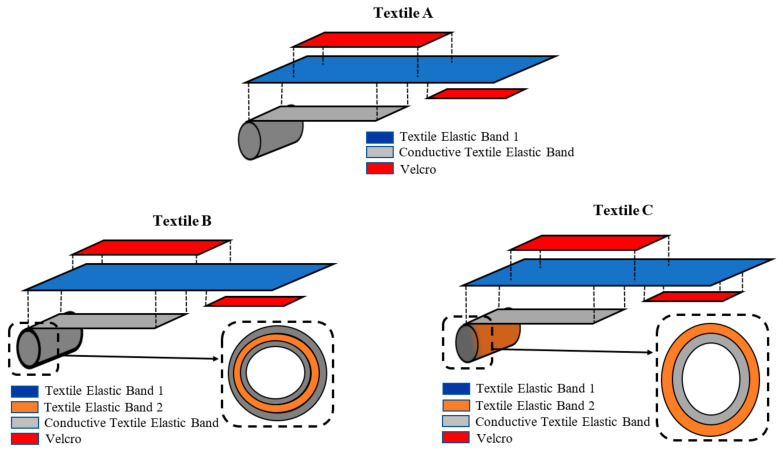
Schematic representation of the textile electrodes analyzed in this paper. All the electrodes consisted of a bracelet fabricated by combining textile elastic materials and the conductive fabric (Shielded Technik-tex P180 + B by Statex). The textiles referred to as “Textile Elastic Band 1” and “Textile Elastic Band 2” differed in their thicknesses and stretchability as follows: Textile Elastic Band 1 was thicker and more stretchable than Textile Elastic Band 2.

**Figure 3:**
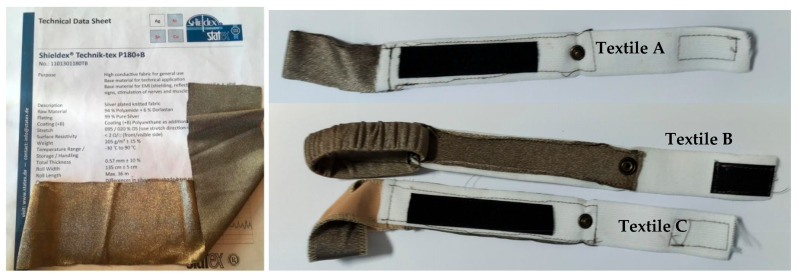
Photographs of the fabricated textile electrodes.

The electrodes analyzed in this paper are illustrated in Figure 2, and a photograph of them is shown in Figure 3. As can be seen, all the analyzed electrodes were designed to form bracelets with heights of 2 cm (Textile B) and 3 cm (Textile A and Textile C). As per the adopted materials, elastic conductive and non-conductive materials were adopted. All electrodes consisted of two elastic bracelets: an internal conductive bracelet attached to an external non-conductive bracelet. The outer bracelet consisted of an elastic band (Elastic Band 1, see Figure 2) which closed with adhesive Velcro. It acted as an insulator and allowed the electrode to be fixed firmly on the wrist by ensuring a good fit, regardless of the size of the wrist.

The electrode referred to as Textile A was the one analyzed in [24] with an optimized implementation so as to improve its fit. In this case, the inner bracelet consisted of only the conductive fabric around which the non-conductive band with a Velcro closure was wrapped.

The electrodes referred to as Textile B and Textile C differed for the conductive fabric bracelet. For the electrode of Textile B, the conductive fabric had an elastic textile core (Elastic Band 2, see Figure 2) and the conductive fabric covered the elastic but was not sewn to it. Finally, for the electrode of Textile C, the conductive fabric was sewn to an elastic band (Elastic Band 2, see Figure 2) so to cover the side of the elastic in contact with the skin.

The basic idea behind the developed electrodes was the investigation of various designs with the aim of optimizing comfort and facilitating the application of the electrodes by non-expert users.

The conductive fabric used was Shielded Technik-tex P180 + B by Statex [26]. It is a knitted fabric that is stretchable in one direction and has the following parameters:

- raw material: 94% polyamide +6% Dorlastan
- total thickness: 0.57 mm ± 10%
- metal plated: 99.9% pure silver
- surface resistivity (both sides): <2 Ω/☐
- stretch: 095/020% OS (one stretch direction)
- temperature range: −30 °C–90 °C

This fabric is particularly suitable for fabricating electrodes for measuring vital parameters since it allows for obtaining a homogeneous current distribution; in this regard, the use of silver to make the fabric conductive makes it suitable for direct application to the skin. Furthermore, this fabric can be sewn, washed, and ironed, similar to common non-conductive fabrics.

As can be seen from Figure 2 and Figure 3, all bracelets had a snap button which allowed them to be attached to a shirt. The button could be used to connect the electrodes with a conductive wire which could be used for measurements. Using a small bioimpedance meter, this solution would allow the system (electrodes and impedance meter) to be integrated into a shirt.

## 3. Results

For each PUT, four measurements were performed: one with the Ag/AgCl disposable electrodes produced by EF Medica Ltd. and one for each textile electrode (Textile A, Textile B, and Textile C).

The commercial electrodes had a solid adhesive gel with high conductivity, which allowed for minimizing the contact resistance without requiring specific treatments for the skin (i.e., application of additional gels). As per the textile electrodes, all measurements were performed without adding electrolytic gel or other substances to improve the adhesion/contact with the skin. 

Each PUT measurement with the different electrodes was performed consecutively, without any break between one measurement and the next except for the time needed to apply the electrodes (a few seconds).

**Figure 4:**
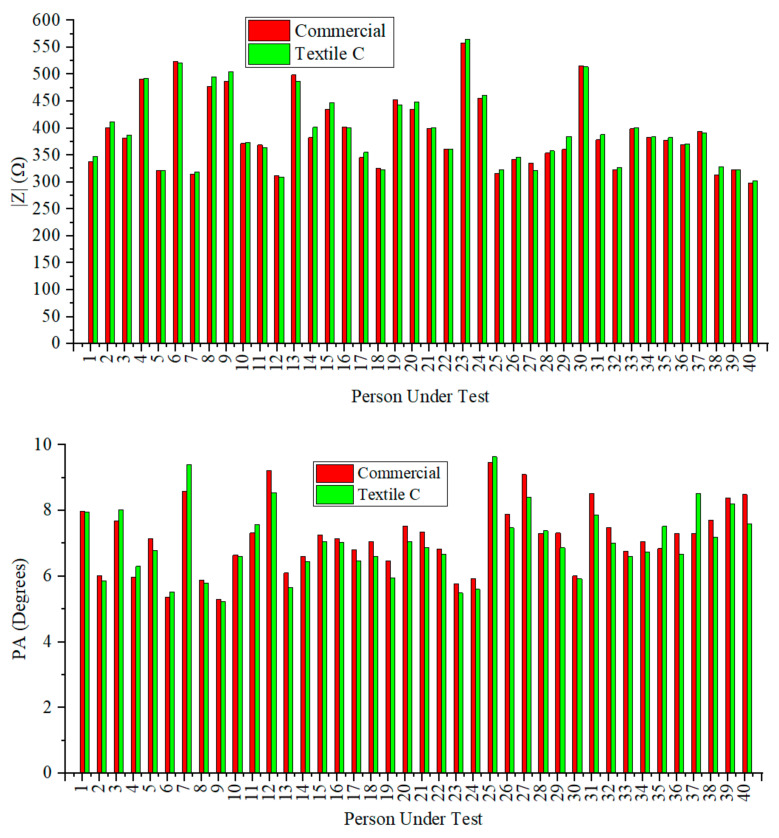
Comparison of the values obtained for |*Z*| and PA by using the Ag/AgCl commercial electrodes and the Textile C electrodes.

A comparison between the values obtained for the module (|*Z*|) and the phase angle (PA) of the impedance with the commercial and textile electrodes is provided in Figure 4. Measurements with textile electrodes refer to Textile C (similar results were obtained for Textile A and Textile B). As can be seen, a good agreement between the values measured with commercial and textile electrodes was obtained for all the PUTs.

In order to validate the feasibility of using textile electrodes, the Passing–Bablok regression method [27] was adopted for comparing the data provided by the textile electrodes with those corresponding to the commercial electrodes. This method was first proposed in 1983 as a method for testing the agreement between two sets of measurements obtained via different systems [27]. In this paper, the Passing–Bablok regression analysis was exploited to investigate the presence of a bias (constant error) or/and a proportional error between the set of data provided by the commercial electrodes and that obtained with the textile electrodes.

**Figure 5:**
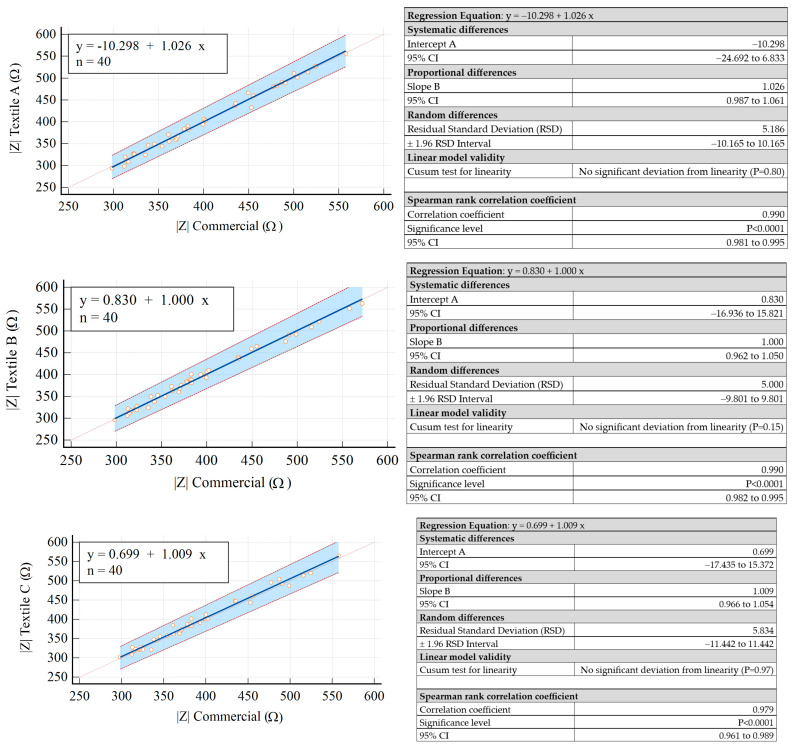
Passing–Bablok regression analysis results obtained for |*Z*|. The thin dotted line is the line for identity (y = x) while the thick blue line is the line for best fit. The red dashed lines indicate the 95% confidence intervals.

**Figure 6:**
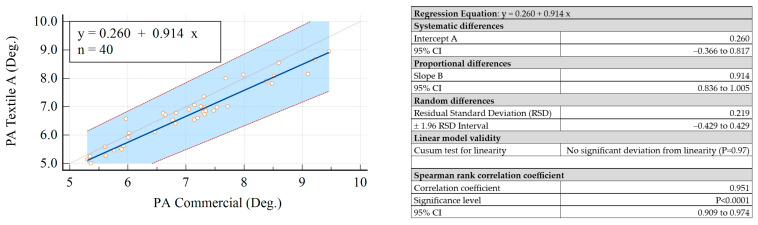

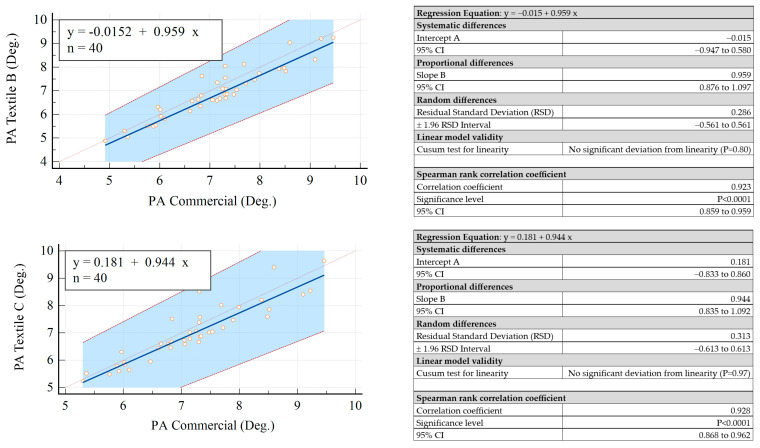
Passing–Bablok regression analysis results obtained for PA. The thin dotted line is the line for identity (y = x) while the thick blue line is the line for best fit. The red dashed lines indicate the 95% confidence intervals.

The achieved results are summarized in Figure 5 for |*Z*| and in Figure 6 for PA.

According to the results obtained for the 95% confidence intervals (95% CI) for both the intercept and slope, it could be concluded that there was no constant difference between the measured data obtained with commercial electrodes and those obtained with the textile electrodes.

It can therefore be concluded that the measured data provided by the textile electrodes had a linear relationship with those provided by the commercial electrodes, and they did not present significant differences, i.e., the two types of electrodes were interchangeable. Additionally, it was important to observe that the random differences obtained for all electrodes were compatible with the accuracy of the measurement setup (see the results summarized in Figure 5 and Figure 6). It was also observed that the three solutions of textile electrodes provided very close results. The best performance in terms of variability of the measured values with respect to the commercial electrodes was provided by Textile B.

Compared to previous solutions of textile electrodes that have been proposed in recent reference literature, the electrodes analyzed in this paper appeared to be an excellent alternative both for their ease of use and their performance and stability of the measurements. However, it was difficult to make an analytical comparison since, in most cases, the comparisons with commercial electrodes were limited to measurements from few subjects (and thus, they were not sufficient for drawing conclusions).

In particular, according to the comparison with commercial electrodes, the electrodes presented in this paper provided results similar to those obtained with the electrodes proposed in [23] and fabricated by using the Silitex P130 fabric by Statex. In this regard, however, it is important to note that the comparison carried out in this paper exploited measurements from 40 PUTs while the one developed in [23] referred to measurements from only 15 PUTs, which was a decidedly lower number. However, some considerations could be made on the ease of use of the two electrodes. During the measurements performed using the electrodes proposed in [23], the authors experienced the need to pay attention to the application of the electrodes, trying to place them in a position with few hairs. In other words, it was verified that the presence of hair affected the measurements both in terms of *R* and *X*. The bracelet shape of the electrodes proposed in this paper and the elastic fabric (Shielded Technik-tex P180 + B) by Statex allowed us to overcome this problem.

In fact, the measurements performed on the 40 subjects did not reveal a dependence of the measurements on the presence of hair (considering that most of the persons under test were men); rather, the electrodes could be worn as a simple bracelet, thus facilitating their application by non-expert users.

Finally, it is worth observing that the forty volunteers who participated in the experimental tests were Italian men and women aged between 19 and 26 years. Although it was believed that this aspect (ethnicity and age of the participants) did not influence the behavior of the electrodes, it could be appropriate to extend the study to other volunteers of different ethnicities and age ranges.

### 3.1. Measurement Repeatability

The variability of measurements over time was investigated through the measurements from PUT 1 (see Table 1). Ten sets of measurements were performed with a 5 min break between one set of measurements and the next. Each measurement set consisted of four measurements (one for each electrode). The person under test remained in a sitting position for the whole time of the measurement campaign.

**Figure 7:**
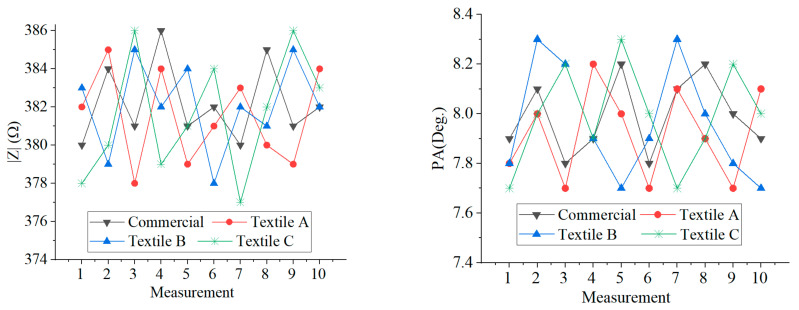
Results of the ten measurements performed on PUT 1.

The achieved results are summarized in Figure 7, where the data obtained with the textile electrodes are compared with those obtained with the commercial ones. As can be seen, similar results were obtained for all the electrodes. When comparing the repeated measurements over time, small differences were observed for both |*Z*| and PA for all electrodes.

### 3.2. Performance after Washing

The performance of the textile electrodes after washing was investigated, and measurements were performed on PUT 1.

**Figure 8:**
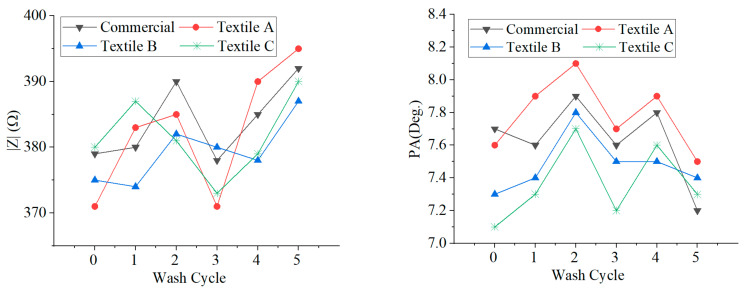
Investigation of the performance of the textile electrodes after washing.

In particular, five washing cycles were performed, and measurements were taken after each washing cycle. The electrodes were hand-washed in cold water (30–37 °C) and left to dry for a day at room temperature. The obtained results are illustrated in Figure 8. Given the need to let the electrodes dry, the measurements were performed on different days. As can be seen, the electrodes appeared to keep working properly even after five washing cycles.

### 3.3. Comfort

The comfort related to the use of the electrodes was investigated. Ten PUTs (five women and five men) were asked to wear each bracelet for one hour, with a half-hour break between one trial and the next.

After wearing all the bracelets, the PUTs were asked to rate the bracelets on a scale from one to give with respect to: (1) ease of wearing of the bracelets, and (2) comfort.

The results of the questionnaire are summarized in Table 2. As can be seen, the results obtained for Textiles B and C were very similar and were slightly better for Textile B, which was evaluated as being easier to wear and more comfortable.

As for Textile A, almost all PUTs rated it as being uncomfortable to wear. As reported by the PUTs, in Textiles B and C, the presence of the Textile Elastic Band 2 (see Figure 2), which acted as a support for the conductive elastic fabric, made it easier to wear. The support helped the adherence of the conductive elastic fabric around the wrist, which, in Textile A, tended to roll up, forming a cord.

## 4. Conclusions

Wristband electrodes for hand-to-hand bioimpedance measurements were investigated. Three different implementations of bracelet electrodes were analyzed through measurements from 40 healthy individuals. All the analyzed electrodes were fabricated using a combination of non-conductive elastic textile materials and a conductive fabric. The conductive fabric was Shielded Technik-tex P180 + B by Statex, and it allowed for obtaining a homogeneous current distribution. In particular, all the analyzed textile electrodes consisted of an internal conductive elastic bracelet fixed to an external non-conductive one, which served to stably fix the electrode to the wrist. The three implementations differed for the conductive bracelet, and different designs were investigated in order to maximize the comfort and ease of application of the electrodes.

Measurements at 50 kHz were performed, and the data provided by the textile electrodes were compared with those obtained with the Ag/AgCl commercial electrodes. The results demonstrated a very high correlation for both the module and the phase angle measured with textile and commercial electrodes. In particular, the Passing–Bablok regression method was exploited to estimate the agreement between the data obtained with the textile electrodes and those provided by the commercial ones. The achieved results demonstrated that the measured data provided by the textile electrodes did not present significant differences with respect to those obtained with the commercial electrodes.

In this regard, it is important to note that the applications that exploit bioimpedance measurements analyze the evolution of bioimpedance measurements over time rather than the value of a single measurement. In most cases, the objective is to analyze how the body composition evolves over time in order to evaluate, for example, the effects of a diet or a therapy. Accordingly, in view of the results obtained from the comparisons with the commercial electrodes for both the measurements from different individuals and those from the same individual over time, the proposed electrodes appeared to be an excellent alternative to commercial electrodes for implementing a wearable system for bioimpedance measurements.

## Figures and Tables

**Table 1:** PUTs’ characteristics.

	Age (Years)	Height (cm)	Weight (kg)	Gender		Age (Years)	Height (cm)	Weight (kg)	Gender
PUT 1	25	176	80	Male	PUT 21	19	175	65	Male
PUT 2	24	182	77.5	Male	PUT 22	23	180	93	Male
PUT 3	26	172	68	Male	PUT 23	25	170	63	Female
PUT 4	19	172	53	Male	PUT 24	26	172	73	Female
PUT 5	19	194	82	Male	PUT 25	24	171	69	Male
PUT 6	24	160	49	Female	PUT 26	25	175	71	Male
PUT 7	24	179	74	Male	PUT 27	24	181	91	Male
PUT 8	32	181	90	Male	PUT 28	20	175	75	Male
PUT 9	24	170	52	Female	PUT 29	21	175	65	Male
PUT 10	25	173	72	Male	PUT 30	25	160	55	Female
PUT 11	21	174	66	Male	PUT 31	26	177	82	Male
PUT 12	21	180	85	Male	PUT 32	25	186	82	Male
PUT 13	21	190	75	Male	PUT 33	25	175	83	Male
PUT 14	22	165	63	Female	PUT 34	25	177	73	Male
PUT 15	21	161	63	Female	PUT 35	24	180	76	Male
PUT 16	22	170	61	Male	PUT 36	18	185	65	Male
PUT 17	21	180	80	Male	PUT 37	19	174	70	Male
PUT 18	23	182	90	Male	PUT 38	20	175	75	Male
PUT 19	19	162	75	Female	PUT 39	18	180	97	Male
PUT 20	19	165	60	Male	PUT 40	19	190	117	Male

**Table 2:** Comfort questionnaire results.

	Easy to Use			Comfort		
	Textile A	Textile B	Textile C	Textile A	Textile B	Textile C
**PUT 1**	5	5	5	5	5	5
**PUT 2**	3	5	4	4	4	5
**PUT 3**	4	5	5	4	5	5
**PUT 4**	3	5	4	5	4	5
**PUT 5**	2	5	4	4	5	4
**PUT 6**	3	5	5	3	5	4
**PUT 7**	4	5	5	4	5	5
**PUT 8**	3	4	5	4	5	4
**PUT 9**	5	5	5	5	5	5
**PUT 10**	4	5	5	4	5	5
**Average score**	**3.6**	**4.9**	**4.7**	**4.2**	**4.8**	**4.7**

## Data Availability

Not applicable.
